# Association of the admission albumin-hemoglobin index with postoperative outcomes in older adults with hip fracture: a *post-hoc* analysis of a prospective study

**DOI:** 10.3389/fnut.2026.1948773

**Published:** 2026-10-28

**Authors:** Hairui Liang, Kangzu Peng, Bingyu Li, Yuhao Kan, Bo Li, Gang Liu, Xinbao Wu, Minghui Yang

**Affiliations:** 1 Department of Orthopaedics and Traumatology, Beijing Jishuitan Hospital, Capital Medical University, Beijing, China; 2 National Center for Orthopaedics, Beijing, China

**Keywords:** albumin–hemoglobin index, all-cause mortality, health-related quality of life, hip fracture, older adults

## Abstract

**Background:**

Older adults with hip fracture are at high risk of postoperative mortality and impaired health-related quality of life. The albumin–hemoglobin index (AHI) combines nutritional and anemia-related information, but its prognostic value remains unclear. We evaluated the associations of admission AHI with 3-year all-cause mortality and postoperative health-related quality of life.

**Methods:**

Patients aged ≥65 years who underwent hip fracture surgery between November 2018 and November 2019 were prospectively enrolled. AHI was calculated from admission serum albumin and hemoglobin levels and analyzed continuously, by quartiles, and using an exploratory cutoff. The primary outcome was 3-year all-cause mortality; the secondary outcome was longitudinal change in EQ-5D utility. Kaplan–Meier analysis, Cox models, restricted cubic splines, linear mixed-effects models, and LASSO Cox regression were used.

**Results:**

Among 1,092 patients screened, 947 were included. Lower AHI was associated with older age, lower body mass index, poorer prefracture mobility, and worse overall health status. Three-year survival differed significantly across quartiles, with the lowest survival in Q1 and the highest in Q4. Each 1,000-unit increase in AHI was associated with a 43% lower mortality risk (adjusted hazard ratio [HR], 0.57; 95% confidence interval [CI], 0.47–0.70; *P* < 0.001). Compared with Q1, the adjusted HRs for Q2, Q3, and Q4 were 0.69 (95% CI, 0.48–0.98), 0.45 (95% CI, 0.28–0.70), and 0.28 (95% CI, 0.16–0.49), respectively. Patients with AHI >4673 had lower mortality than those with AHI ≤ 4673 (HR, 0.46; 95% CI, 0.32–0.66; *P* < 0.001). In the multivariable-adjusted restricted cubic spline analysis, the overall association was significant (*P* for overall <0.001), with no evidence of nonlinearity (*P* for nonlinearity = 0.855). Higher AHI was also associated with more favorable EQ-5D recovery, and LASSO sensitivity analysis supported the primary findings.

**Conclusions:**

Lower admission AHI was independently associated with higher 3-year mortality and poorer recovery in health-related quality of life after hip fracture surgery. AHI may provide a simple tool for perioperative risk stratification and individualized management.

## 1 Introduction

Hip fracture is one of the most serious fragility fractures in older adults and is commonly associated with impaired mobility, reduced quality of life, an increased need for long-term care, and a higher risk of mortality (1, 2). With population aging, the healthcare and societal burden of hip fracture continues to increase (3). Despite advances in timely surgery, orthogeriatric co-management, fracture liaison services, and standardized rehabilitation, postoperative outcomes in older adults with hip fracture remain suboptimal (4, 5). Therefore, the early identification of simple, reliable, and potentially modifiable prognostic indicators is important for perioperative risk stratification, individualized management, and improvement of long-term outcomes (6).

Older adults with hip fracture commonly have multiple chronic diseases, frailty, impaired mobility, inadequate nutritional reserves, and anemia (7). Previous studies have shown that both malnutrition and anemia are associated with postoperative complications, limited functional recovery, prolonged hospital stays, and increased mortality risk (8, 9). Although nutrition-related indicators, including serum albumin, the prognostic nutritional index, and the geriatric nutritional risk index, have been used for risk assessment in older adults with hip fracture, these measures primarily focus on nutritional reserves, inflammation, or weight-related risk and do not directly incorporate information on anemia and oxygen-carrying capacity reflected by hemoglobin levels (10, 11). Low albumin and low hemoglobin levels may jointly indicate reduced physiological reserve, impaired tolerance to surgical stress, and limited rehabilitation potential; therefore, their combined assessment may facilitate the identification of patients at higher risk of adverse outcomes (12).

The albumin–hemoglobin index (AHI) is a novel composite measure that integrates serum albumin and hemoglobin levels, both of which are routinely assessed at hospital admission. It is readily available, inexpensive, and clinically interpretable. Previous studies in gastrointestinal and other surgical populations have reported associations between AHI and postoperative complications and mortality, suggesting its potential prognostic value (13). More recently, AHI has also been investigated in older adults with hip fracture, with preoperative AHI reported to be associated with 1-year mortality (14). However, the available evidence remains limited. Previous studies have primarily focused on short-term or 1-year mortality, and evidence regarding longer-term mortality is scarce. Moreover, the goals of hip fracture treatment extend beyond reducing mortality, as postoperative functional recovery and health-related quality of life are also important outcomes for older patients and their families. The association between AHI and postoperative health-related quality of life has not yet been adequately evaluated.

This study used a prospective cohort of older adults with hip fracture to evaluate the associations of admission AHI with 3-year all-cause mortality and postoperative recovery in health-related quality of life. By analyzing AHI as both a continuous and categorical variable and combining survival analysis, dose–response assessment, and longitudinal analysis of health-related quality of life, we aimed to clarify the clinical value of AHI for long-term prognostic assessment and to provide evidence for early risk stratification and perioperative management in this population.

## 2 Methods

### 2.1 Study design and participants

This study was a *post hoc* analysis of a prospective cohort of older adults with hip fracture. The original cohort study was conducted at Beijing Jishuitan Hospital between November 2018 and November 2019 to evaluate perioperative management and long-term postoperative outcomes in older adults with hip fracture and was registered at ClinicalTrials.gov (Identifier: NCT03184896) (15). The study protocol was approved by the Biomedical Ethics Committee of Beijing Jishuitan Hospital (Approval No. 201807-11), and written informed consent was obtained from all participants or their legally authorized representatives. The study was conducted in accordance with the principles of the Declaration of Helsinki.

Patients aged ≥65 years who were hospitalized for hip fracture and underwent surgical treatment were eligible for inclusion. Hip fractures included femoral neck fractures and intertrochanteric fractures. Surgical treatment was selected according to fracture type and individual clinical characteristics. Femoral neck fractures were treated with internal fixation, hemiarthroplasty, or total hip arthroplasty as clinically appropriate, whereas intertrochanteric fractures were treated with intramedullary fixation. Serum albumin and hemoglobin measurements at admission were required. The main exclusion criteria were pathological fracture, terminal malignancy, and missing admission serum albumin or hemoglobin data. A total of 947 patients were included in the final analysis. The patient selection process is shown in Figure 1.

**Figure 1:**
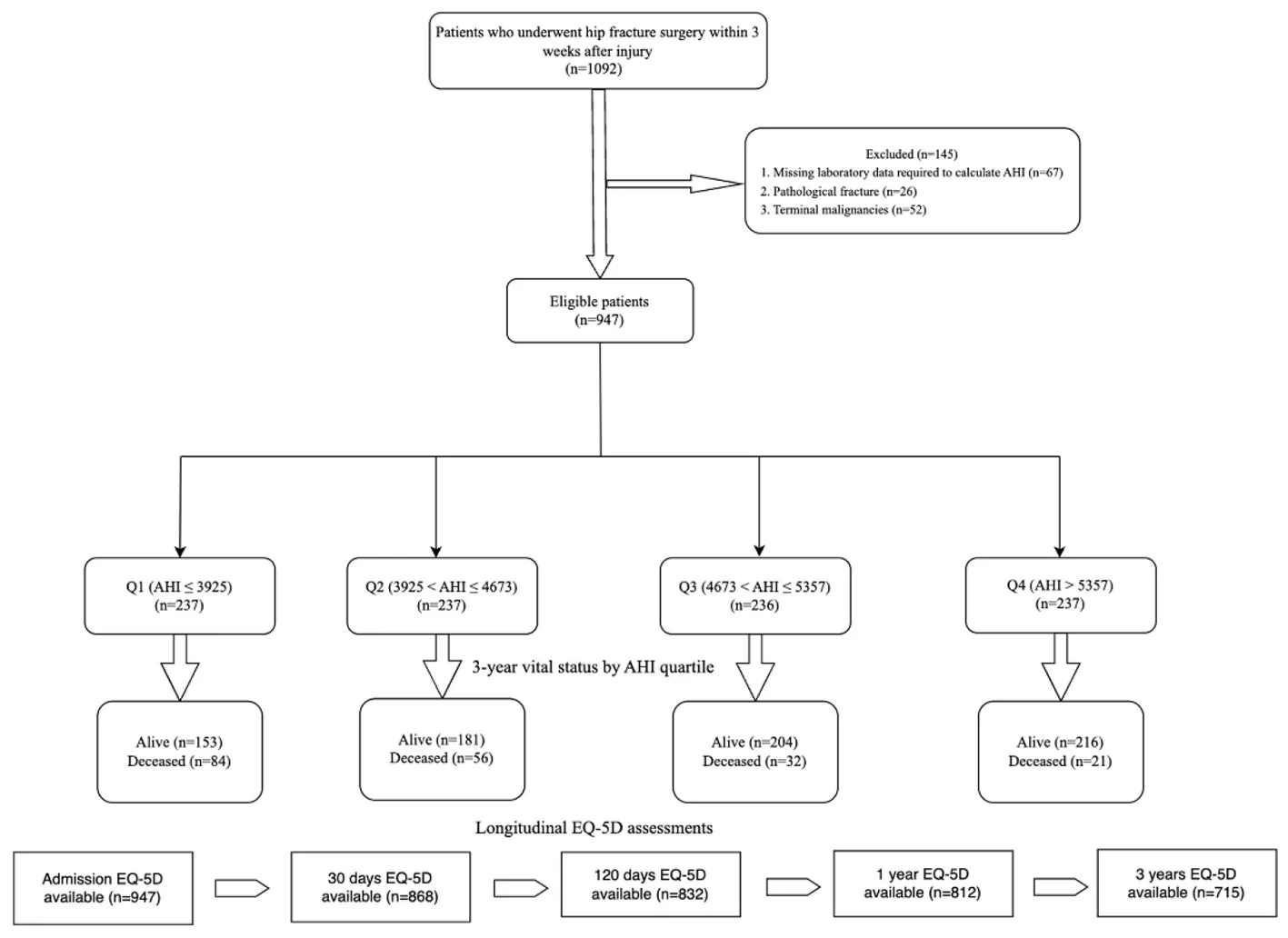
Flowchart of patient selection and grouping according to admission AHI quartiles.

### 2.2 Calculation and categorization of AHI

Peripheral venous blood samples for serum albumin and hemoglobin were routinely obtained during the initial emergency department assessment as part of the standardized fast-track pathway for older adults with hip fracture. The first available measurements after presentation were used to calculate AHI and were generally obtained before blood transfusion, substantial fluid resuscitation, or other interventions likely to materially affect albumin or hemoglobin concentrations. The albumin–hemoglobin index (AHI) was calculated using the following formula: AHI = serum albumin (g/L) × hemoglobin (g/L).

Patients were categorized into four groups according to the quartile distribution of AHI: Q1 (≤ 3925), Q2 (>3925 to ≤ 4673), Q3 (>4673 to ≤ 5357), and Q4 (>5357). In the mortality analyses, AHI was entered into the models as a continuous variable, a quartile-based categorical variable, and a dichotomous variable. In the continuous analysis, AHI was modeled per 1,000-unit increase. For the exploratory dichotomous analysis, the sample median AHI of 4673, corresponding to the boundary between Q2 and Q3, was used to divide patients into lower-AHI (≤ 4673; Q1–Q2) and higher-AHI (>4673; Q3–Q4) groups. This cutoff was based on the sample distribution rather than an independently identified threshold from the RCS analysis or an outcome-optimized cutoff selection procedure.

### 2.3 Data collection

Demographic characteristics, clinical features, comorbidities, fracture-related information, and perioperative variables were prospectively collected. Demographic variables included age, sex, and body mass index (BMI). Clinical characteristics and comorbidities included diabetes mellitus, heart disease, hypertension, stroke, dementia, depression, Parkinson disease, osteoporosis, visual impairment, cognitive impairment, Charlson Comorbidity Index (CCI), American Society of Anesthesiologists (ASA) physical status classification, smoking status, and current drinking status. Osteoporosis was clinically ascertained based on a low-energy mechanism of injury, a documented history of osteoporosis, and DXA findings when available; DXA confirmation was not required in all patients. Social and functional variables included educational level, living alone, living at home, housebound status, pre-fracture mobility, number of falls, and whether the injury resulted from a non-ground-level fall.

Fracture-related variables included fracture type, categorized as femoral neck fracture or intertrochanteric fracture. Perioperative variables included time from injury to surgery (TIS), TIS > 48 h, length of hospital stay (LOS), and prolonged LOS. Admission serum albumin, hemoglobin, and left ventricular ejection fraction (LVEF) were also recorded. LVEF was included as a baseline marker of cardiac function and reserve potentially relevant to perioperative risk.

### 2.4 Follow-up and outcome measures

All patients were followed up at 30 days, 120 days, 1 year, and 3 years after surgery. Follow-up assessments were conducted primarily through outpatient visits or telephone interviews. Trained orthopedic surgeons or research personnel collected information on survival status and health-related quality of life.

The primary outcome was 3-year all-cause mortality after surgery. All-cause mortality was defined as death from any cause during follow-up. Survival time was calculated from the date of surgery to the date of death or the last valid follow-up.

The secondary outcome was health-related quality of life at different follow-up time points, assessed using the EuroQol 5-Dimension 5-Level questionnaire (EQ-5D-5L) (16). The EQ-5D-5L comprises five dimensions: mobility, self-care, usual activities, pain/discomfort, and anxiety/depression, with five severity levels for each dimension. EQ-5D-5L health states were converted into EQ-5D utility values using the Chinese population value set, with higher scores indicating better health-related quality of life (17).

### 2.5 Statistical analysis

Continuous variables are presented as the mean ± standard deviation or median (interquartile range), according to their distributions, whereas categorical variables are presented as numbers and percentages. Baseline characteristics across AHI quartiles were compared using one-way analysis of variance, the Kruskal–Wallis test, the chi-square test, or Fisher's exact test, as appropriate.

Kaplan–Meier curves were generated to estimate 3-year survival across AHI quartiles, and between-group differences were assessed using the log-rank test. Cox proportional hazards regression models were subsequently used to evaluate the association between AHI and 3-year all-cause mortality. Results are reported as hazard ratios (HRs) with 95% confidence intervals (CIs).

Restricted cubic spline (RCS) analysis was performed to assess the continuous association and potential non-linearity between admission AHI and 3-year all-cause mortality.

Linear mixed-effects models were used to evaluate differences in EQ-5D utility values across AHI quartiles during follow-up. Results are presented as model estimates with 95% CIs.

To further assess the robustness of the findings, least absolute shrinkage and selection operator (LASSO) regression was used for covariate selection. The optimal penalty parameter, λ, was selected through cross-validation, and variables with non-zero coefficients were included in the LASSO-selected model as a sensitivity analysis for the Cox regression results.

All statistical analyses were performed using R software (version 4.4.2; R Foundation for Statistical Computing, Vienna, Austria; http://www.R-project.org). All statistical tests were two-sided, and a *P* value <0.05 was considered statistically significant.

## 3 Results

### 3.1 Baseline characteristics

A total of 947 older adults with hip fracture were included in the final analysis. According to the quartile distribution of admission albumin–hemoglobin index (AHI), patients were categorized into Q1 (≤ 3925, *n* = 237), Q2 (>3925 to ≤ 4673, *n* = 237), Q3 (>4673 to ≤ 5357, *n* = 236), and Q4 (>5357, *n* = 237). The patient selection and grouping process is shown in Figure 1.

Baseline characteristics differed significantly across AHI quartiles. Compared with patients in the higher AHI groups, those in the lower AHI groups were older, had a lower body mass index, were more likely to require mobility assistance before fracture, and had a higher proportion of intertrochanteric fractures. Regarding comorbidities and overall clinical status, the lower AHI groups had higher proportions of heart disease, ASA grade ≥III, housebound status, and cognitive impairment. The prevalence of heart disease decreased from 40.51% in Q1 to 25.74% in Q4 (*P* = 0.005), and the proportion of patients with ASA grade ≥III was highest in Q1 (46.84%, *P* = 0.016). Significant between-group differences were also observed in diabetes, educational level, current drinking status, and sex distribution.

Regarding perioperative variables, patients in the lower AHI groups had a longer time from injury to surgery (TIS) and a longer hospital stay. The median TIS was 3.30 days (interquartile range [IQR], 2.13–5.58) in Q1 and 2.73 days (IQR, 1.94–3.89) in Q4 (*P* < 0.001). The median length of hospital stay was 5.67 days (IQR, 4.46–6.96) in Q1 and 4.79 days (IQR, 3.83–5.92) in Q4 (*P* < 0.001). No significant difference in left ventricular ejection fraction was observed across the four groups (*P* = 0.773). Complete baseline characteristics are presented in Table 1.

**Table 1:** Baseline characteristics according to admission AHI quartiles.

Variables	Total (*n* = 947)	Q1 (≤ 3925, *n* = 237)	Q2 (3925– ≤ 4673, *n* = 237)	Q3 (4673– ≤ 5357, *n* = 236)	Q4 (>5357, *n* = 237)	*P* value
Age, mean ± SD, years	79.59 ± 7.81	83.08 ± 6.93	80.69 ± 7.44	78.47 ± 7.58	76.14 ± 7.57	<0.001
Female, n (%)	686 (72.44)	180 (75.95)	173 (73.00)	181 (76.69)	152 (64.14)	0.008
BMI, mean ± SD, kg/m^2^	22.96 ± 3.86	21.98 ± 4.15	22.47 ± 3.72	23.74 ± 3.84	23.67 ± 3.43	<0.001
Assisted mobility, n (%)	279 (29.46)	91 (38.40)	72 (30.38)	60 (25.42)	56 (23.63)	0.002
Fracture type, n (%)						<0.001
FNF	484 (51.11)	44 (18.57)	101 (42.62)	143 (60.59)	196 (82.70)	
ITF	463 (48.89)	193 (81.43)	136 (57.38)	93 (39.41)	41 (17.30)	
LVEF, median (IQR), %	65.98 (63.35, 68.00)	65.59 (63.00, 68.00)	65.98 (63.00, 68.00)	65.99 (64.00, 67.91)	65.99 (64.00, 67.39)	0.773
Heart disease, n (%)	327 (34.53)	96 (40.51)	89 (37.55)	81 (34.32)	61 (25.74)	0.005
DM, n (%)	277 (29.25)	65 (27.43)	60 (25.32)	66 (27.97)	86 (36.29)	0.046
Dementia, n (%)	55 (5.81)	16 (6.75)	13 (5.49)	9 (3.81)	17 (7.17)	0.399
Hypertension, n (%)	611 (64.52)	161 (67.93)	152 (64.14)	158 (66.95)	140 (59.07)	0.179
Stroke, n (%)	246 (25.98)	58 (24.47)	56 (23.63)	74 (31.36)	58 (24.47)	0.188
Depression, n (%)	19 (2.01)	7 (2.95)	7 (2.95)	1 (0.42)	4 (1.69)	0.110
Parkinson disease, n (%)	26 (2.75)	5 (2.11)	7 (2.95)	10 (4.24)	4 (1.69)	0.340
Osteoporosis, n (%)	917 (96.83)	228 (96.20)	225 (94.94)	234 (99.15)	230 (97.05)	0.064
Visual impairment, n (%)	388 (40.97)	94 (39.66)	103 (43.46)	96 (40.68)	95 (40.08)	0.835
Cognitive impairment, n (%)	103 (10.88)	31 (13.08)	35 (14.77)	20 (8.47)	17 (7.17)	0.022
CCI, n (%)						0.158
0	315 (33.26)	69 (29.11)	87 (36.71)	79 (33.47)	80 (33.76)	
1	340 (35.90)	87 (36.71)	91 (38.40)	75 (31.78)	87 (36.71)	
2	190 (20.06)	47 (19.83)	36 (15.19)	56 (23.73)	51 (21.52)	
≥3	102 (10.77)	34 (14.35)	23 (9.70)	26 (11.02)	19 (8.02)	
Education, n (%)						0.005
Illiterate	180 (19.01)	57 (24.05)	49 (20.68)	42 (17.80)	32 (13.50)	
Primary or lower	244 (25.77)	67 (28.27)	73 (30.80)	54 (22.88)	50 (21.10)	
High school	254 (26.82)	57 (24.05)	56 (23.63)	63 (26.69)	78 (32.91)	
University	269 (28.41)	56 (23.63)	59 (24.89)	77 (32.63)	77 (32.49)	
ASA grade, n (%)						0.016
I	115 (12.14)	17 (7.17)	31 (13.08)	32 (13.56)	35 (14.77)	
II	476 (50.26)	109 (45.99)	120 (50.63)	126 (53.39)	121 (51.05)	
≥III	356 (37.59)	111 (46.84)	86 (36.29)	78 (33.05)	81 (34.18)	
Ever/current smoker, n (%)	158 (16.68)	37 (15.61)	35 (14.77)	41 (17.37)	45 (18.99)	0.613
Current drinker, n (%)	44 (4.65)	5 (2.11)	10 (4.22)	11 (4.66)	18 (7.59)	0.042
Living alone, n (%)	107 (11.30)	20 (8.44)	34 (14.35)	30 (12.71)	23 (9.70)	0.158
Living at home, n (%)	383 (40.44)	100 (42.19)	104 (43.88)	86 (36.44)	93 (39.24)	0.365
Housebound, n (%)	224 (23.65)	70 (29.54)	61 (25.74)	48 (20.34)	45 (18.99)	0.024
No. of falls, n/N (%)						0.164
0	385/936 (41.13)	101/236 (42.80)	94/234 (40.17)	101/233 (43.35)	89/233 (38.20)	
1	425/936 (45.41)	93/236 (39.41)	110/234 (47.01)	109/233 (46.78)	113/233 (48.50)	
≥2	126/936 (13.46)	42/236 (17.80)	30/234 (12.82)	23/233 (9.87)	31/233 (13.30)	
Non-ground-level fall, n/N (%)	103/937 (10.99)	28/236 (11.86)	25/235 (10.64)	21/233 (9.01)	29/233 (12.45)	0.648
TIS, median (IQR), days	3.02 (2.04, 4.54)	3.30 (2.13, 5.58)	3.21 (2.25, 4.74)	2.85 (1.94, 4.03)	2.73 (1.94, 3.89)	<0.001
TIS >48 h, n (%)	718 (75.82)	189 (79.75)	192 (81.01)	170 (72.03)	167 (70.46)	0.012
LOS, median (IQR), days	5.00 (4.00, 6.42)	5.67 (4.46, 6.96)	5.12 (4.12, 6.75)	4.75 (3.96, 5.89)	4.79 (3.83, 5.92)	<0.001
Prolonged LOS, n (%)	465 (49.10)	143 (60.34)	132 (55.70)	91 (38.56)	99 (41.77)	<0.001

Values are presented as mean ± standard deviation, median (interquartile range), number (percentage), or number/available number (percentage), as appropriate. *P* values were calculated using one-way analysis of variance, Kruskal–Wallis test, chi-square test, or Fisher's exact test, as appropriate. AHI quartiles were defined as follows: Q1, ≤ 3925; Q2, 3925– ≤ 4673; Q3, 4673– ≤ 5357; Q4, >5357. AHI, albumin–hemoglobin index; ASA, American Society of Anesthesiologists; BMI, body mass index; CCI, Charlson Comorbidity Index; DM, diabetes mellitus; FNF, femoral neck fracture; IQR, interquartile range; ITF, intertrochanteric fracture; LOS, length of hospital stay; LVEF, left ventricular ejection fraction; SD, standard deviation; TIS, time from injury to surgery.

### 3.2 Association between AHI and 3-year all-cause mortality

Kaplan–Meier analysis showed clear separation of the 3-year survival curves across AHI quartiles, with the lowest survival probability in Q1 and the highest in Q4 (Figure 2).

**Figure 2:**
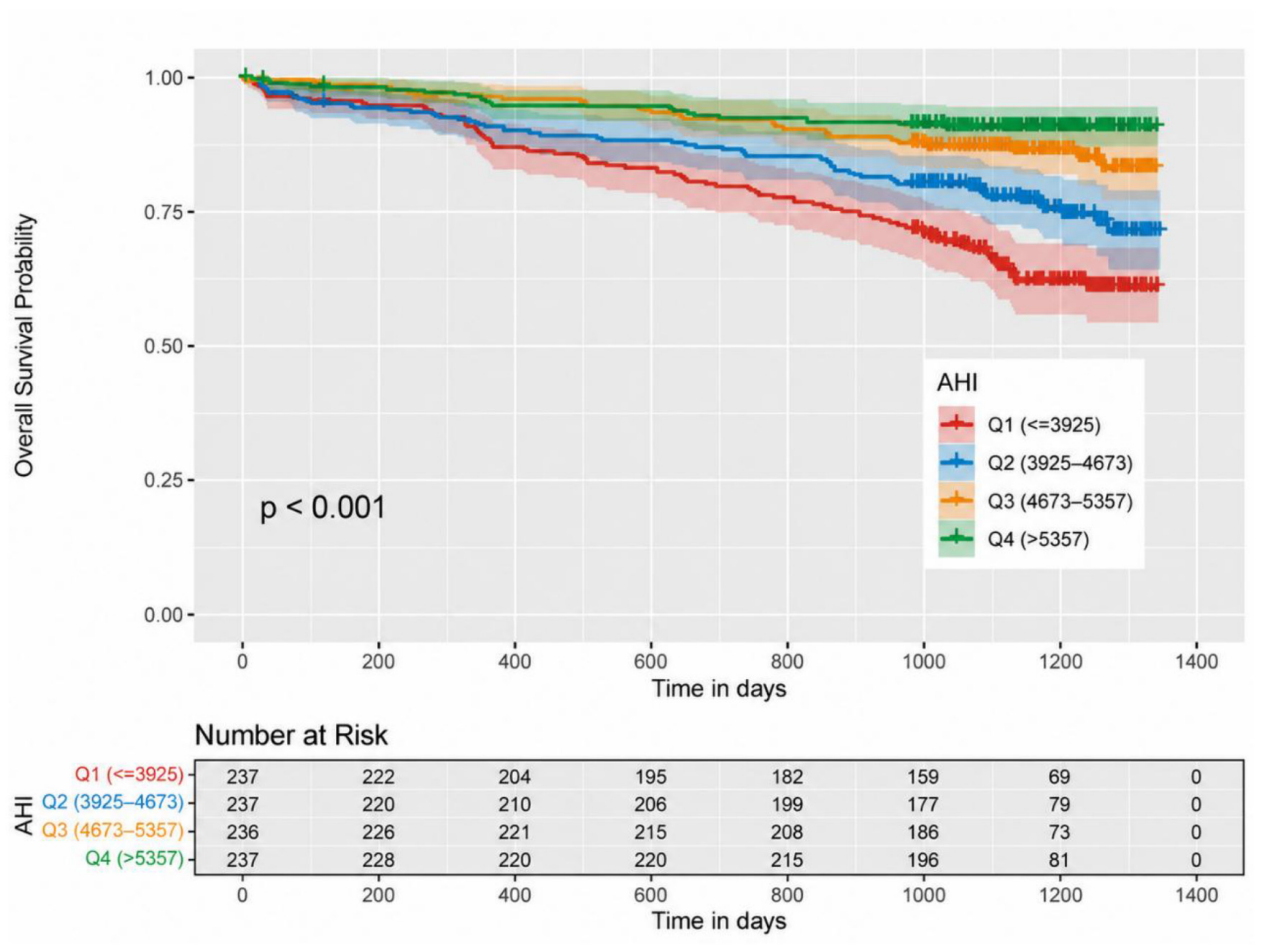
Kaplan–Meier survival curves for 3-year all-cause mortality according to admission AHI quartiles.

Cox proportional hazards models demonstrated a significant association between AHI and 3-year all-cause mortality. When modeled as a continuous variable, each 1,000-unit increase in AHI was associated with a significantly lower risk of 3-year mortality. This association remained significant in the unadjusted model, Model 1, and the primary multivariable-adjusted Model 2. In Model 2, the HR per 1,000-unit increase in AHI was 0.57 (95% CI, 0.47–0.70; *P* < 0.001).

Using Q1 as the reference group, higher AHI quartiles were associated with progressively lower risks of 3-year mortality. In Model 2, the adjusted HRs for Q2, Q3, and Q4 were 0.69 (95% CI, 0.48–0.98; *P* = 0.039), 0.45 (95% CI, 0.28–0.70; *P* < 0.001), and 0.28 (95% CI, 0.16–0.49; *P* < 0.001), respectively. In the sensitivity model based on LASSO-selected covariates, the results were generally consistent with those of the primary multivariable model, and Q3 and Q4 remained associated with significantly lower risks of 3-year all-cause mortality. The LASSO variable-selection process and the variables retained in the final model are shown in Supplementary Figure S1.

In the exploratory median-based dichotomous analysis, patients with AHI >4673 had a lower risk of 3-year all-cause mortality than those with AHI ≤ 4673 (adjusted HR, 0.46; 95% CI, 0.32–0.66; *P* < 0.001). The Cox regression results for the association between AHI and 3-year all-cause mortality are presented in Table 2.

**Table 2:** Association between AHI and 3-year mortality in older patients with hip fractures.

AHI variable type	Admission AHI	Unadjusted		Model 1^a^		Model 2^b^		Model 3^c^	
		HR (95% CI)	*P* value	HR (95% CI)	*P* value	HR (95% CI)	*P* value	HR (95% CI)	*P* value
**Continuous**	Per 1000-unit increase	0.52 (0.44~0.61)	<0.001	0.62 (0.52~0.73)	<0.001	0.57 (0.47~0.70)	<0.001	—	—
**Quartiles**	Q1 (≤ 3925)	1 (Reference)		1 (Reference)		1 (Reference)		1 (Reference)	
	Q2 (3925– ≤ 4673)	0.63 (0.45~0.88)	0.007	0.72 (0.51~1.01)	0.059	0.69 (0.48~0.98)	0.039	0.71 (0.49~1.01)	0.059
	Q3 (4673– ≤ 5357)	0.35 (0.23~0.52)	<0.001	0.47 (0.31~0.72)	<0.001	0.45 (0.28~0.70)	<0.001	0.43 (0.27~0.68)	<0.001
	Q4 (>5357)	0.22 (0.14~0.36)	<0.001	0.34 (0.21~0.56)	<0.001	0.28 (0.16~0.49)	<0.001	0.26 (0.15~0.46)	<0.001
**Dichotomy** ^§^	Lower (≤ 4673)	1 (Reference)		1 (Reference)		1 (Reference)		—	—
	Higher (>4673)	0.35 (0.26~0.48)	<0.001	0.48 (0.35~0.67)	<0.001	0.46 (0.32~0.66)	<0.001	—	—

^**a**^Model 1, adjusted for age, sex and BMI. ^**b**^Model 2, adjusted for Model 1 + diabetes, housebound, cognitive impairment, type of fracture, ASA, mobility, heart disease, current drinker, time from injury to surgery and educational level. ^**c**^Model 3, adjusted for covariates selected by least absolute shrinkage and selection operator (LASSO) regression (Supplementary Figure). ^§^Dichotomy, AHI was dichotomized at the sample median (4673) for exploratory analysis, corresponding to the boundary between Q2 and Q3. AHI, albumin–hemoglobin index; CI, confidence interval; HR, hazard ratio.

### 3.3 Restricted cubic spline analysis of the association between AHI and 3-Year mortality

Restricted cubic spline analysis was performed to further evaluate the association between AHI, modeled as a continuous variable, and 3-year all-cause mortality. Both the unadjusted and multivariable-adjusted RCS curves showed an overall decline in the risk of 3-year mortality with increasing AHI. In both analyses, AHI was significantly associated with 3-year mortality risk overall (both *P* for overall <0.001), with no evidence of a non-linear association in either the unadjusted model (*P* for non-linearity = 0.669) or the multivariable-adjusted model (*P* for non-linearity = 0.855). The RCS results are shown in Figure 3.

**Figure 3:**
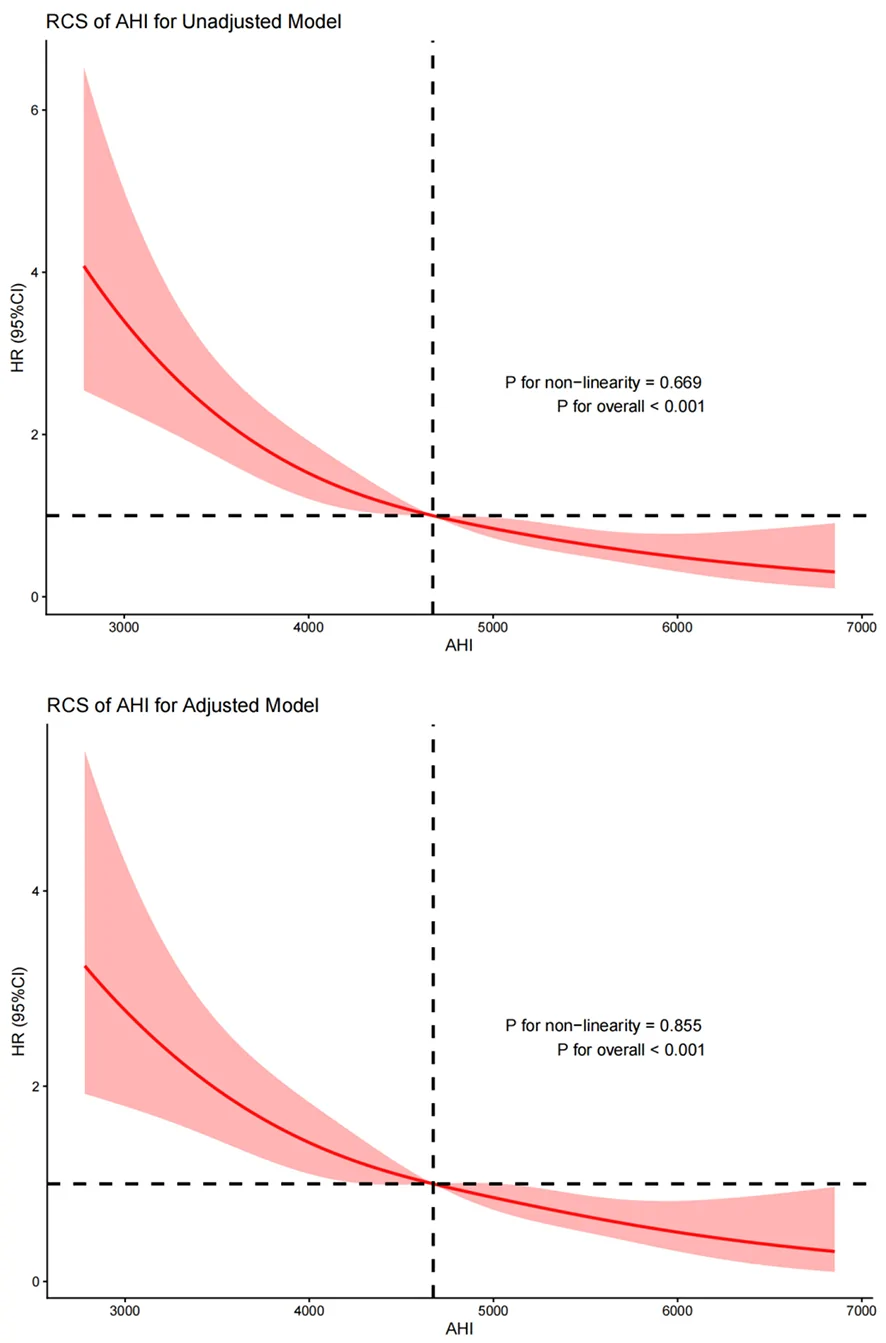
Unadjusted and adjusted restricted cubic spline analyses of the association between admission AHI and 3-year all-cause mortality. The vertical dashed line indicates the sample median AHI (4673), which was used as the reference value (HR=1).

### 3.4 Association between AHI and postoperative EQ-5D recovery

Changes in postoperative EQ-5D utility values across AHI quartiles are shown in Figure 4. Overall, patients in Q1 had the lowest EQ-5D utility values during follow-up, whereas those in the higher AHI quartiles showed more favorable recovery patterns. At admission, EQ-5D utility values were similar across the four AHI quartiles; however, between-group differences became apparent at 30 days, 120 days, 1 year, and 3 years after surgery.

**Figure 4:**
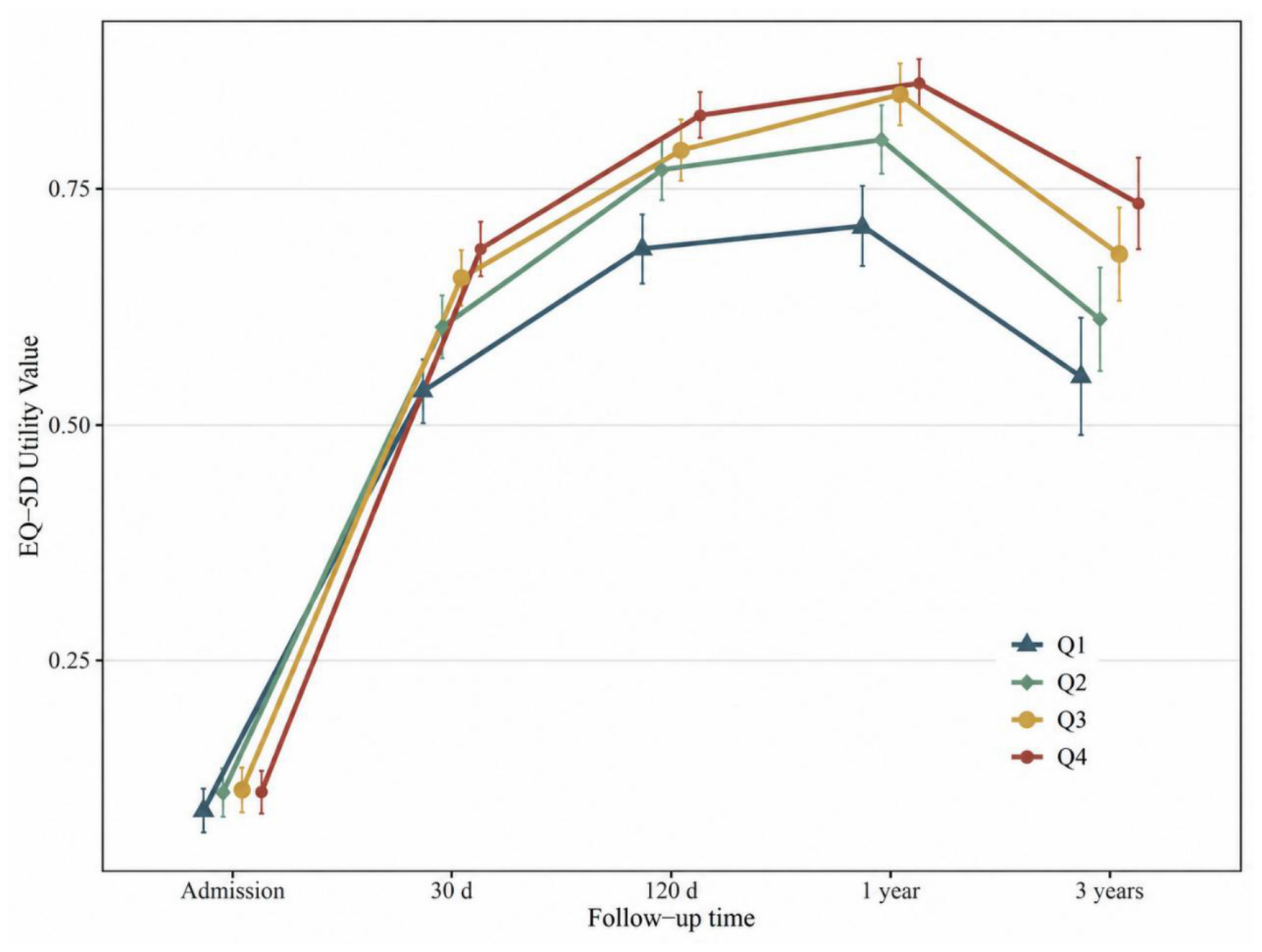
Longitudinal changes in mean EQ-5D utility according to admission AHI quartiles. Error bars indicate 95% confidence intervals.

Linear mixed-effects models showed no significant differences between Q1 and the other AHI quartiles at admission. After multivariable adjustment, significant differences were observed between Q1 and Q2, Q3, and Q4 at 30 days, 120 days, and 1 year after surgery. At 3 years, Q3 and Q4 remained significantly different from Q1, whereas the difference between Q2 and Q1 did not reach statistical significance (*P* = 0.075). The complete results of the linear mixed-effects models are presented in Supplementary Table S1.

## 4 Discussion

Hip fracture is one of the most serious fragility fractures in older adults. Although care models such as orthogeriatric co-management, multidisciplinary collaboration, standardized rehabilitation, and fracture liaison services have been increasingly implemented, postoperative mortality, functional limitation, and the need for long-term care remain substantial (7, 18–21). Therefore, the early identification of high-risk patients using routinely available clinical indicators is important for perioperative risk stratification and individualized management. Routine hematologic and biochemical tests are readily available and relatively inexpensive. Against this background, the present study used a prospective cohort of older adults with hip fracture to systematically evaluate the associations of admission albumin–hemoglobin index (AHI) with 3-year all-cause mortality and postoperative health-related quality of life, thereby assessing its potential value for long-term prognostic evaluation, early risk stratification, and individualized perioperative management.

Previous studies have shown that older adults with hip fracture face high risks of mortality and functional dependence, particularly those of advanced age or with a high comorbidity burden or cognitive impairment (22, 23). In a study of older nursing home residents with hip fracture in the United States, Neuman et al. reported a 180-day mortality rate of 36.2%, with more than half of the patients experiencing either death or new-onset complete dependence in locomotion; advanced age, comorbidity burden, and cognitive impairment were associated with adverse outcomes (24). Similarly, patients in the lower AHI groups in the present study were more likely to be older and to have poorer pre-fracture mobility, cognitive impairment, and a greater burden of comorbidities. These findings suggest that AHI may reflect not only nutritional and anemia-related status but also the overall physiological reserve and vulnerability of older adults with hip fracture.

Malnutrition and anemia are common and interrelated perioperative problems in older adults with hip fracture (25). Previous studies have shown that malnutrition is associated with postoperative complications, impaired functional recovery, adverse in-hospital outcomes, and increased mortality, and nutrition-related indicators such as serum albumin, the prognostic nutritional index, and the geriatric nutritional risk index have been used for risk assessment in this population (10, 26). Similarly, anemia at admission has been associated with higher risks of postoperative complications and mortality after hip fracture (27). Serum albumin primarily reflects nutritional reserve, inflammatory burden, and hepatic synthetic capacity, whereas hemoglobin reflects anemia status, oxygen-carrying capacity, and tolerance to trauma and surgical stress (28). In older adults with hip fracture, low albumin and low hemoglobin may respectively indicate nutrition- and inflammation-related risk and reduced oxygen-carrying capacity due to anemia; when present together, they may reflect diminished physiological reserve, poorer tolerance of perioperative stress, and limited rehabilitation potential (29). The rationale for evaluating AHI in the present study was to integrate these two clinically relevant dimensions into a single, readily available measure, rather than to assume that their mathematical combination necessarily provides superior predictive performance compared with albumin or hemoglobin individually. Accordingly, AHI may be viewed as a simple composite marker of nutritional and anemia-related risk.

Evidence regarding the use of AHI in older adults with hip fracture remains limited. A recent retrospective study by Choi et al. showed that preoperative AHI was associated with 1-year mortality and had potential prognostic value in this population (14). Our findings are consistent with the overall direction of that study, indicating that lower AHI is associated with a higher risk of mortality. However, the present study extends the existing evidence by using a prospective cohort, evaluating 3-year all-cause mortality, and confirming the robustness of the association through multivariable adjustment, restricted cubic spline analysis, and LASSO-based sensitivity analysis. In addition, the inclusion of EQ-5D outcomes suggests that the clinical relevance of AHI may extend beyond mortality risk assessment to postoperative recovery in health-related quality of life.

Our findings showed that each 1,000-unit increase in AHI was associated with a significantly lower risk of 3-year all-cause mortality after multivariable adjustment. Compared with the lowest AHI quartile, patients in the higher AHI quartiles, particularly Q3 and Q4, had markedly lower mortality risks. Restricted cubic spline analysis further demonstrated a significant overall association between AHI and 3-year mortality, with no evidence of non-linearity, suggesting a continuous and gradual risk gradient rather than a distinct threshold or inflection point. Therefore, the value of 4673 should be interpreted as an exploratory cutoff for risk stratification rather than a validated clinical threshold. Differences in postoperative EQ-5D outcomes were also observed across AHI groups. Patients with lower AHI showed less favorable recovery patterns in health-related quality of life at multiple follow-up time points, whereas those with higher AHI generally had higher EQ-5D utility values. However, these longitudinal findings should be interpreted with caution because later EQ-5D assessments were limited to surviving patients. Although the linear mixed-effects models used all available repeated measurements, mortality-related informative missingness could not be fully addressed. Previous studies have shown that hip fracture can lead to persistent functional decline and mobility limitations, with many patients failing to regain their pre-fracture level of function (30, 31). Our findings further suggest that the combined nutritional and anemia-related status at admission may be associated with postoperative recovery in health-related quality of life. This is clinically relevant because the goals of hip fracture care should extend beyond reducing mortality to include promoting functional recovery, improving quality of life, and reducing the need for long-term care.

The present findings suggest that AHI, a simple index calculated from admission serum albumin and hemoglobin levels, is readily available and inexpensive and may facilitate early risk identification in older adults with hip fracture. Patients with low AHI may represent a high-risk subgroup characterized by poorer nutritional status, anemia, and reduced overall physiological reserve; therefore, AHI may provide a useful reference for perioperative monitoring, nutritional assessment, anemia management, comprehensive geriatric evaluation, and individualized rehabilitation planning. In light of previous evidence that orthogeriatric co-management can improve selected outcomes after hip fracture, AHI may serve as a practical risk-stratification tool within integrated care pathways. By using a prospective cohort with 3-year follow-up, evaluating both mortality and longitudinal changes in EQ-5D utility, and applying multiple analytical approaches, this study provides a more comprehensive assessment of the association between AHI and clinically relevant long-term outcomes in older adults with hip fracture.

This study has several limitations. First, this was a *post hoc* analysis of a single-center prospective cohort; therefore, the external generalizability of the findings should be further validated in multicenter cohorts, and the AHI cutoff derived from the sample median should not be regarded as a clinically validated threshold. Second, despite multivariable adjustment and LASSO-based sensitivity analysis, residual confounding cannot be completely excluded, including potential confounding related to specific surgical procedures that were not separately adjusted for; therefore, the observed association should not be interpreted as fully independent or causal. Third, AHI was derived from the first available admission albumin and hemoglobin measurements obtained during the initial emergency assessment, and perioperative changes were not evaluated. Although these measurements were generally obtained before major interventions, some influence of initial stabilization cannot be excluded. In addition, the incremental prognostic value of AHI beyond albumin, hemoglobin, or other nutritional indicators was not directly assessed and warrants further investigation. Finally, the long-term EQ-5D assessment may have been affected by loss to follow-up and selective missingness related to mortality.

## 5 Conclusion

This study demonstrated that lower admission AHI was independently associated with an increased risk of 3-year all-cause mortality and poorer recovery in health-related quality of life among older adults with hip fracture. Calculated from routinely measured admission serum albumin and hemoglobin levels, AHI may serve as a simple composite risk marker to complement perioperative risk stratification and inform nutritional assessment, anemia management, and individualized rehabilitation planning. Further multicenter studies are warranted to validate its external applicability, clinically relevant cutoff values, and incremental prognostic value.

## Data Availability

The data analyzed in this study is subject to the following licenses/restrictions: The datasets generated and/or analyzed during the current study are not publicly available due to privacy and ethical restrictions, but are available from the corresponding author on reasonable request. Requests to access these datasets should be directed to Minghui Yang, doctyang0125@126.com.
