# Supplementary material for: Association of the admission albumin-hemoglobin index with postoperative outcomes in older adults with hip fracture: a post-hoc analysis of a prospective study
Source: Front Nutr. 2026 Oct 28;13:1948773. doi: 10.3389/fnut.2026.1948773 (PMC13752031; doi:10.3389/fnut.2026.1948773)
Supplement: Supplementary file 1 [file Table_1.docx]

Supplementary Table S1：Unadjusted and adjusted linear mixed-effects models for EQ-5D utility across admission AHI quartiles

| Follow-up time | AHI quartile | Unadjusted model estimate (95% CI) | P value | Adjusted model estimate (95% CI) | P value |
| --- | --- | --- | --- | --- | --- |
| Admission | Q1 | Reference | — | Reference | — |
|  | Q2 | -0.0192 (-0.065 to 0.026) | 0.407 | -0.0038 (-0.048 to 0.041) | 0.868 |
|  | Q3 | -0.0223 (-0.068 to 0.023) | 0.336 | -0.0084 (-0.040 to 0.020) | 0.846 |
|  | Q4 | -0.0199 (-0.065 to 0.025) | 0.390 | -0.0133 (-0.040 to 0.020) | 0.583 |
| 30 days | Q1 | Reference | — | Reference | — |
|  | Q2 | -0.0677 (-0.115 to -0.021) | 0.005 | -0.0531 (-0.099 to -0.007) | 0.024 |
|  | Q3 | -0.1220 (-0.170 to -0.074) | <0.001 | -0.0935 (-0.142 to -0.045) | <0.001 |
|  | Q4 | -0.1521 (-0.199 to -0.105) | <0.001 | -0.1194 (-0.169 to -0.070) | <0.001 |
| 120 days | Q1 | Reference | — | Reference | — |
|  | Q2 | -0.0861 (-0.134 to -0.038) | <0.001 | -0.0712 (-0.118 to -0.024) | 0.003 |
|  | Q3 | -0.1100 (-0.158 to -0.062) | <0.001 | -0.0811 (-0.130 to -0.032) | 0.001 |
|  | Q4 | -0.1438 (-0.192 to -0.096) | <0.001 | -0.1110 (-0.161 to -0.061) | <0.001 |
| 365 days | Q1 | Reference | — | Reference | — |
|  | Q2 | -0.0954 (-0.144 to -0.046) | <0.001 | -0.0776 (-0.126 to -0.030) | 0.002 |
|  | Q3 | -0.1433 (-0.192 to -0.094) | <0.001 | -0.1145 (-0.164 to -0.065) | <0.001 |
|  | Q4 | -0.1580 (-0.207 to -0.109) | <0.001 | -0.1250 (-0.176 to -0.074) | <0.001 |
| 3 years | Q1 | Reference | — | Reference | — |
|  | Q2 | -0.0628 (-0.116 to -0.009) | 0.030 | -0.0478 (-0.101 to 0.005) | 0.075 |
|  | Q3 | -0.1355 (-0.189 to -0.082) | 0.001 | -0.1117 (-0.165 to -0.058) | <0.001 |
|  | Q4 | -0.1914 (-0.244 to -0.139) | <0.001 | -0.1605 (-0.215 to -0.106) | <0.001 |

**Note:** Values are model estimates from linear mixed-effects models and are presented as originally parameterized in the statistical output, with Q1 as the reference group. Q1, ≤3925; Q2, 3925–≤4673; Q3, 4673–≤5357; Q4, >5357. The adjusted model included age, sex, body mass index, diabetes, housebound status, cognitive impairment, fracture type, ASA grade, pre-fracture mobility, heart disease, current drinking status, time from injury to surgery, and educational level. AHI, albumin–hemoglobin index; ASA, American Society of Anesthesiologists; CI, confidence interval; EQ-5D, EuroQol five-dimension questionnaire.

**Supplementary Figure S1. LASSO variable selection for 3-year all-cause mortality.**
(A) Coefficient profile plot of candidate predictors in the LASSO Cox regression model.
(B) Cross-validation curve for selecting the optimal penalty parameter λ.
(C) Variables retained in the final LASSO model and their corresponding coefficients. AHI group was retained among the selected predictors, supporting the robustness of its association with 3-year all-cause mortality.

AHI, albumin–hemoglobin index; ASA, American Society of Anesthesiologists; BMI, body mass index; CCI, Charlson Comorbidity Index; LASSO, least absolute shrinkage and selection operator; LVEF, left ventricular ejection fraction; TIS, time from injury to surgery.
